# Alzheimer's disease biomarker discovery using *in silico* literature mining and clinical validation

**DOI:** 10.1186/1479-5876-10-217

**Published:** 2012-10-31

**Authors:** Ines Greco, Nicola Day, Joanna Riddoch-Contreras, Jane Reed, Hilkka Soininen, Iwona Kłoszewska, Magda Tsolaki, Bruno Vellas, Christian Spenger, Patrizia Mecocci, Lars-Olof Wahlund, Andrew Simmons, Julie Barnes, Simon Lovestone

**Affiliations:** 1 King’s College London, Institute of Psychiatry, De Crespigny Park, London, SE5 8AF, UK; 2 BioWisdom Ltd, Cambridge, UK (now Instem Scientific; 3 University of Eastern Finland and University Hospital of Kuopio, Kuopio, Finland; 4 Medical University of Lodz, Lodz, Poland; 5 3rd Department of Neurology, "G. Papanicolaou" Hospital, Aristotle University of Thessaloniki, Thessaloniki, Greece; 6 UMR INSERM 1027, Gerontopole, CHU Toulouse, University of Toulouse, Toulouse, France; 7 Department of Clinical Science, Intervention and Technology, Karolinska Institutet, Stockholm, Sweden; 8 Institute of Gerontology and Geriatrics, University of Perugia, Perugia, Italy; 9 Department of Neurobiology, Care Sciences and Society, Karolinska Institutet, Stockholm, Sweden; 10 Currently at Somaxa Ltd and Abcodia Ltd, London, UK

**Keywords:** Alzheimer’s disease, Proteomics, Biomarkers, Choline acetyltransferase (ChAt), Urokinase-type plasminogen activator receptor (PLAUR), Intelligence network, Bioinformatics, MRI, *in silico*, Literature mining

## Abstract

**Background:**

Alzheimer’s Disease (AD) is the most widespread form of dementia in the elderly but despite progress made in recent years towards a mechanistic understanding, there is still an urgent need for disease modification therapy and for early diagnostic tests. Substantial international efforts are being made to discover and validate biomarkers for AD using candidate analytes and various data-driven 'omics' approaches. Cerebrospinal fluid is in many ways the tissue of choice for biomarkers of brain disease but is limited by patient and clinician acceptability, and increasing attention is being paid to the search for blood-based biomarkers. The aim of this study was to use a novel *in silico* approach to discover a set of candidate biomarkers for AD.

**Methods:**

We used an *in silico* literature mining approach to identify potential biomarkers by creating a summarized set of assertional metadata derived from relevant legacy information. We then assessed the validity of this approach using direct assays of the identified biomarkers in plasma by immunodetection methods.

**Results:**

Using this *in silico* approach, we identified 25 biomarker candidates, at least three of which have subsequently been reported to be altered in blood or CSF from AD patients. Two further candidate biomarkers, indicated from the *in silico* approach, were choline acetyltransferase and urokinase-type plasminogen activator receptor. Using immunodetection, we showed that, in a large sample set, these markers are either altered in disease or correlate with MRI markers of atrophy.

**Conclusions:**

These data support as a proof of concept the use of data mining and *in silico* analyses to derive valid biomarker candidates for AD and, by extension, for other disorders.

## Background

Alzheimer’s disease (AD) is one of the commonest causes of dementia resulting in a severe loss of intellectual abilities including memory. The main histological features of AD in brain are amyloid plaques and neurofibrillary tangles, due to accumulation, respectively, of amyloid beta (AÎ²) peptide and tau protein in insoluble form. The causes of this, and other pathology found in the AD brain, are not known with certainty but are likely to be multifactorial. This multifactorial and only partially understood pathogenesis complicates both drug and biomarker discovery.

The search for biomarkers to aid accurate diagnosis, predict progression and for use in clinical trials has become a major research goal [1,2]. The most widely used strategy for the discovery of biomarkers is predicated on the identification of potential candidate biomarkers using knowledge of disease processes followed by validation, comparing healthy control to affected subjects [3]. In many respects, the optimal source of human tissue for the investigation of AD biomarkers is cerebrospinal fluid (CSF) and the demonstration of lowered CSF Aβ and raised CSF tau and phosphorylated tau in AD is the prime example of candidate biomarker discovery and validation leading to qualification using an affected case-normal elderly control design [4].

However, all three approaches - candidate discovery, case–control design and the use of CSF have their drawbacks. CSF although ideal in many respects as it bathes the diseased organ, is limited by patient and clinician acceptability, especially for repeated measurements. Affected case versus normal elderly control experimental design is severely limited by the long prodromal phase in AD, meaning many apparently unaffected controls may have substantial occult pathology. Candidate biomarkers are limited by the complex multifactorial pathology in AD meaning that beyond Aβ and tau it becomes difficult to ascertain true candidates. In an effort to overcome the limitations of CSF, we, and others, have pursued, with some early indications of success, markers in plasma that might act as biomarkers of disease [5]. To overcome the problems of occult pathology and to identify biomarkers of potential utility, two broad approaches have been employed; firstly comparing people with prodromal states, for example in mild cognitive impairment (MCI) who progress to AD, to those who do not, and secondly comparing both affected and asymptomatic people by a non-clinical marker of disease such as MRI measures of atrophy or PET measures of amyloid load [6,7]. This latter approach has been described variously as an endophenotype or extreme phenotype design. To mitigate the limitations of the candidate marker approach many have used a range of data-driven technologies including proteomic and genomic platforms and increasingly these datasets are being aggregated for biomarker discovery and understanding pathogenesis; for example in renal disease where over 200 datasets are mined in a web-based application to identify disease associated proteins [8].

Here we report a novel approach to biomarker discovery through the utilization of data-driven biomarker discovery *in silico* followed by validation in blood using automated analysis of structural MRI as an endophenotype measure of disease. The *in silico* study started by generating massive volumes of assertional data represented in the form of an Intelligence Network. Assertions are simple factual representations of statements made in the biomedical literature and other sources. They can be compiled into a semantically consistent form by applying comprehensive vocabularies and lexical matching approaches to yield a navigable database known as an Intelligence Network (IN). For this study, the IN contained assertions relating to proteins expressed in the brain and associated with pathology relevant to AD. These are two important features for an ideal biomarker for AD, assuming that such proteins could be measured in readily accessible fluids such as blood or urine. An *in vitro* study of two putative markers identified by the *in silico* screen - Choline Acetyltransferase (ChAt) and urokinase-type Plasminogen Activator Receptor (PLAUR) - provided evidence supporting the validity of the method suggesting that *in silico* screening for biomarkers in AD and, by extrapolation, other disorders, is a productive approach.

## Methods

### *In silico* discovery of candidate biomarkers

To identify a set of candidate biomarkers for AD, we applied an informatics approach that would enable a comprehensive analysis of a body of information embedded in publicly available literature sources and other information databases relevant to AD. The approach used resulted in the generation of thousands of highly accurate semantically consistent observational facts, known as assertions, which are represented in the form of subject-verb-object constructs and referenced back to the original source(s) e.g. “Amyloid Deposition_IS ASSOCIATED WITH_Alzheimer’s Disease”; “BACE-1_IS INVOLVED IN_Amyloid Formation”; “Syntax in 1_IS EXPRESSED IN_Hippocampus”.

Behind each assertion is a rich vocabulary that renders that assertion semantically consistent with the other assertions of the same nature. For example, the pathology term Amyloid Deposition can be described within the literature in a variety of ways as ‘abnormal deposition of amyloid plaques’, ‘amyloid infiltration’, ‘amyloid deposits’, ‘amyloid protein deposition’ etc. Amyloid Deposition is defined as the preferred ‘concept’, and all other terms link to that concept to generate a semantically consistent IN. All concepts are defined as a Concept Type e.g. in the case of Amyloid Deposition, a Pathological Observation. The result is a comprehensive and unbiased overview of relevant published data relating to AD, expressed in a format that can be readily navigated, searched and analyzed.

#### Scope of the intelligence network

**Figure 1:**
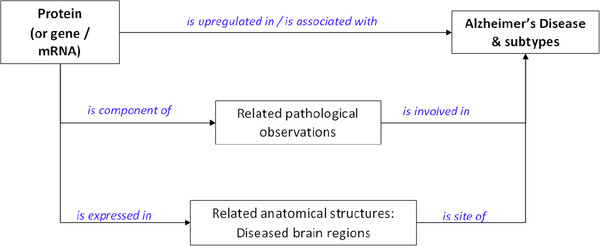
**Network map used to guide the assertion generation for an Intelligence Network relevant for the discovery of candidate biomarkers for AD.** Assertions could be direct links between proteins/mRNAs and AD (and sub-types), or indirect links via gene mutations (e.g. for familial forms of AD, such as OMIM record #104300), via pathological processes (e.g. neuronal degeneration, amyloid formation, neurofibrillary tangles, hippocampal atrophy, tau deposition), or via anatomical structures (e.g. hippocampus, CA3 region, thalamus, temporal lobe). The relations (shown in blue) are examples of the vocabularies used in the assertions.

To define the scope of the IN relevant to the identification of candidate biomarkers of AD, we set the criteria for the ideal biomarker. We defined that any biomarker for AD would ideally be a protein with a known role in the pathological development of the disease and have expression patterns within the brain that correlate with the localized hallmarks of AD pathology. We also defined an interest in proteins that had been reported to show patterns of upregulation. We made assumptions that such proteins could be ultimately identified in accessible fluids such as serum or urine. Thus, the IN that was generated linked data relating to protein/mRNA expression in relevant brain structures (e.g. hippocampus) with relevant pathological observations (e.g. loss of memory and tau phosphorylation). A network map of key Concept Types was then defined (See Figure 1) and this guided the build of the IN.

#### Assertion generation

The derivation of assertions from a body of historic information is challenging for a number of reasons. Firstly, the vocabulary used across the medical literature is highly varied and is typified by the use of synonyms, abbreviations, and idiosyncratic acronyms. Secondly, biomedical writing styles are diverse, with often complex linguistic patterns, involving descriptive terms, extended phrases, and statements or assertions that cross more than one sentence. There has been much effort invested in development of automated text mining methods that yield both sufficiently high accuracy and good recall. Our pragmatic approach is semi-automated i.e. automated extraction of possible subject-verb-object relationships (powered by rich vocabularies and synonym associations) is followed by manual validation.

To enable lexical matching and to deal with the synonym variations across the data sources of interest, a rich set of pre-curated vocabularies was developed, relating to subtypes of AD, AD-related pathological observations, brain regions affected by AD, and proteins (and associated genes/mRNAs). Sample vocabularies used for Pathological Observations and Tissue are illustrated in Table 1. The vocabularies for Protein/mRNAs are too numerous to be listed, but comprised all known human proteins as defined in the publicly accessible protein database UniProt [9]. Each Concept was associated with a comprehensive set of synonymous terms.

**Table 1:** Sample vocabularies for key AD disease, pathological observations, and tissue concepts, used in the AD IN

**Alzheimer's Disease sub-types**	**Pathological observations in AD**	**Brain regions affected in AD**
Alzheimer's Disease	Acute-Phase Reaction	Amygdala
Early Onset Alzheimer's Disease	Amyloid Deposition	Anterior Thalamic Nucleus
Early Onset Familial Alzheimer's Disease	Amyloid Fibril Formation	Basal Nucleus of Meynert
Familial Alzheimer's Disease	Amyloidosis	CA1 region
Incipient Alzheimer's Disease	Asymmetric Cortical Atrophy	CA2 region
Late Onset Alzheimer's Disease	Blood Brain Barrier Dysfunction	CA3 region
Late Onset Sporadic Alzheimer's Disease	Central Nervous System Inflammation	Cholinergic Neuron
Mid-Stage Alzheimer's Disease	Cerebral Atrophy	Diagonal Band of Broca
Mild-to-Moderate Alzheimer's Disease	Cholinergic Dysfunction	Entorhinal Cortex
Moderate Alzheimer's Disease	Corpus Callosum Atrophy	Frontal Lobe
Moderate-to-Severe Alzheimer's Disease	Dystrophic Neuronal Growth	Hippocampus
Sporadic Alzheimer's Disease	Glial Inflammation	Inferior Temporal Gyrus
Severe Alzheimer's Disease	Gliosis	Left Thalamus
	Glucose Hypometabolism	Locus Coeruleus
	Granulovacuolar Degeneration	Medial Temporal Cortex
	Hippocampal Neurodegeneration	Parahippocampal Gyrus
	Inflammation	Parietal Lobe
	Locus Coeruleus Neuronal Loss	Prefrontal Cortex
	Mitochondrial Failure	Septal Nucleus
	Nerve Degeneration	Subiculum
	Neuritic Plaque Formation	Substantia Innominata
	Neurofibrillary Degeneration	Superior Temporal Gyrus
	Neurofibrillary Lesion	Synapse
	Neurofibrillary Tangle Formation	Temporal Isocortex
	Neuroinflammation	Temporal Lobe
	Neuronal Degeneration	Thalamus
	Neuronal Dysfunction	
	Neuronal Dystrophy	
	Neuronal Inclusion Bodies	
	Neuronal Lesion	
	Neuronal Loss	
	Neuronal Necrosis	
	Neuronal Shrinkage	
	Occipital Atrophy	
	Oxidative Damage	
	Oxidative Stress	
	Perivascular Amyloidosis	
	Synapse Dysfunction	
	Synaptic Degeneration	
	Synaptic Loss	
	Synapse Enlargement	
	Tau Deposition	
	Tau Phosphorylation	
	Tau-Mediated Cytotoxicity	

In addition to the use of vocabularies around Proteins, Pathological Observations and Tissues, the assertion-generation procedure also relied on the use of specific verbs or relationships (again with synonyms) between the key Concept Types. The preferred relationships for assertions stating a relationship between AD and various Pathological Observations included ‘AFFECTS’, ‘CAUSES’, ‘HAS FEATURE’, ‘LEADS TO’, ‘RESULTS IN’. For assertions between Anatomical Structure and Protein/mRNA, relationships such as EXPRESSES, HAS CONSTITUENT, IS LOCATION OF, IS SITE OF ALTERATION OF, HAS UPREGULATED were used. For assertions between Protein/mRNA and AD, relationships such as IS AFFECTED IN, IS EXPRESSED IN, IS ALTERED IN, IS COMPONENT OF, IS HIGHER IN, IS INCREASED IN, IS RISK FACTOR FOR, IS LOCATED IN were used.

To support the assertion generation process, a range of publicly accessible information sources were identified. These are detailed in Table 2 and included textual sources, such as full-text literature review papers, Medline abstracts, and reports from a variety of web-based AD research forums, as well as structured databases, such as gene expression databases (NCBI GEO), protein-pathway databases (e.g. GO, KEGG) and protein-disease association databases (e.g. OMIM). All sources provided both vocabularies and relevant assertions. The assertion generation process used the Sofia platform (see http://www.biowisdom.com/tag/sofia/ and [10]) and was powered by the broad vocabularies around Proteins, Observations and Tissues, to enable semantic consistency of all final assertions. All the data sources were accessed between July 2006 and October 2006.

**Table 2:** Data sources used for the generation of the Intelligence Network

**Databases**	**Description**
Alzheimer Disease & Frontotemporal Dementia Mutation Database (http://www.molgen.ua.ac.be/ADMutations/Default.cfm)	The Alzheimer Disease & Frontotemporal Dementia Mutation Database (AD&FTDMDB) aims at collecting all known mutations and non-pathogenic coding variations in the genes related to Alzheimer disease (AD) and frontotemporal dementia (FTD). All data were exported and loaded into Sofia, to create gene-disease assertions.
Diseases Database (http://www.diseasesdatabase.com)	The Diseases database is a cross-referenced medical dictionary of diseases, medications, symptoms, signs and investigations, which was loaded into Sofia and provided assertions linking Alzheimers disease to symptoms and signs, histopathological abnormalities, risk factors etc.
Gene Ontology (http://www.geneontology.org)	The Gene Ontology project provides an ontology of defined terms representing gene product properties. The ontology covers three domains for the gene products: cellular component, molecular function, & biological process. All of GO was processed and loaded into Sofia, and the relevant assertions were then exported into the IN.
Genetic Association Database (http://geneticassociationdb.nih.gov)	The Genetic Association Database is an archive of human genetic association studies of complex diseases and disorders. All the data linking genes to diseases were processed and downloaded into Sofia, and the relevant assertions were then exported into the IN.
Gensat Brain Atlas (http://www.gensat.org)	GENSAT is a gene expression atlas of the developing and adult central nervous system of the mouse. After AD-related brain areas were identified from literature reviews, the relevant genes were exported from GENSAT, and assertions linking gene to anatomical area created and loaded into Sofia.
KEGG (http://www.genome.jp/kegg/pathway.html)	KEGG (Kyoto Encyclopedia of Genes and Genomes) is a bioinformatics resource for linking genomes to life and the environment. Pathways relevant to AD were reviewed, and relevant protein-pathway assertions were generated using Sofia.
NCBI Gene Expression Omnibus (http://www.ncbi.nlm.nih.gov/geo)	Gene Expression Omnibus (GEO) is a database repository of high throughput gene expression data and hybridization arrays, chips, microarrays. GEO was searched for AD-relevant expression data, which were downloaded from the NCBI site and loaded into Sofia.
OMIM (http://www.omim.org/)	Online Mendelian Inheritance in Man (OMIM) is a database that catalogues all the known diseases with a genetic component, and if possible, links them to the relevant genes in the human genome and provides references for further research and tools for genomic analysis of a catalogued gene. All of OMIM Genemap was exported and loaded into Sofia; relevant AD records were used to create gene-disease assertions.
Telemakus knowledgebase (http://www.telemakus.net/AD/)	Telemakus Biomarkers in Alzheimer's Disease & Mild Cognitive Impairment Knowledgebase contains information from AD and MCI biomarker studies. All of the Knowledgebase was exported and loaded into Sofia as protein-disease assertions.
**Textual Data**	**Description**
PubMed (http://www.ncbi.nlm.nih.gov/pubmed/)	PubMed is a service of the U.S. National Library of Medicine that includes over 18 million citations from MEDLINE and other life science journals for biomedical articles back to the 1950s. PubMed contains a rich set of biomedical literature abstracts relevant to many areas. AD-relevant vocabularies within Sofia were used to build a "corpus" of AD-relevant abstracts, which were then used in assertion-generation processes to create disease-protein and disease-process links.
Full text papers and reports	Various full text reviews from journals, and reports from AD websites (Alzheimer Research Forum; http://www.alzforum.org/ and Essential Science Indicators; http://www.esi-topics.com/alzheimer/) were downloaded, and text versions were loaded into Sofia for assertion generation using key AD-related vocabularies.

For unstructured data sources such as Medline, both lexical (pattern-matching) and linguistic (part-of-speech identification) techniques were used to extract relationships that exist between any of the Concepts in the network map. Several repeat extraction methods were applied, each using a different pattern of noun phrase, thus allowing for the diversity of language patterns used. The result was a high level of recall. The extraction procedure yielded a set of “proto-assertions”, composed of subject-verb-object triplets. All proto-assertions were manually checked by trained curators, to ensure that the each component of the triplets had matched appropriately and the assertion was represented accurately in the underlying reference. Inappropriate assertions were discarded. Each curator was able to validate approximately 500 assertions per day. An accuracy level of greater than 97% was confirmed by random sampling and quality control testing (following an ISO 2859 sampling scheme). For structured sources, generally the whole data source was downloaded and parsed into Sofia with the appropriate relationships, and accuracy levels were checked within an appropriate sample set. Overall, the design-build-curate-QC process for the IN took a total of three months. The resulting IN consisted of over 50,000 assertions, linking more than 200 different pathological observation concepts, over 6,500 protein concepts, and over 35 anatomical/tissue concepts.

#### Derivation of candidates from intelligence network

**Figure 2:**
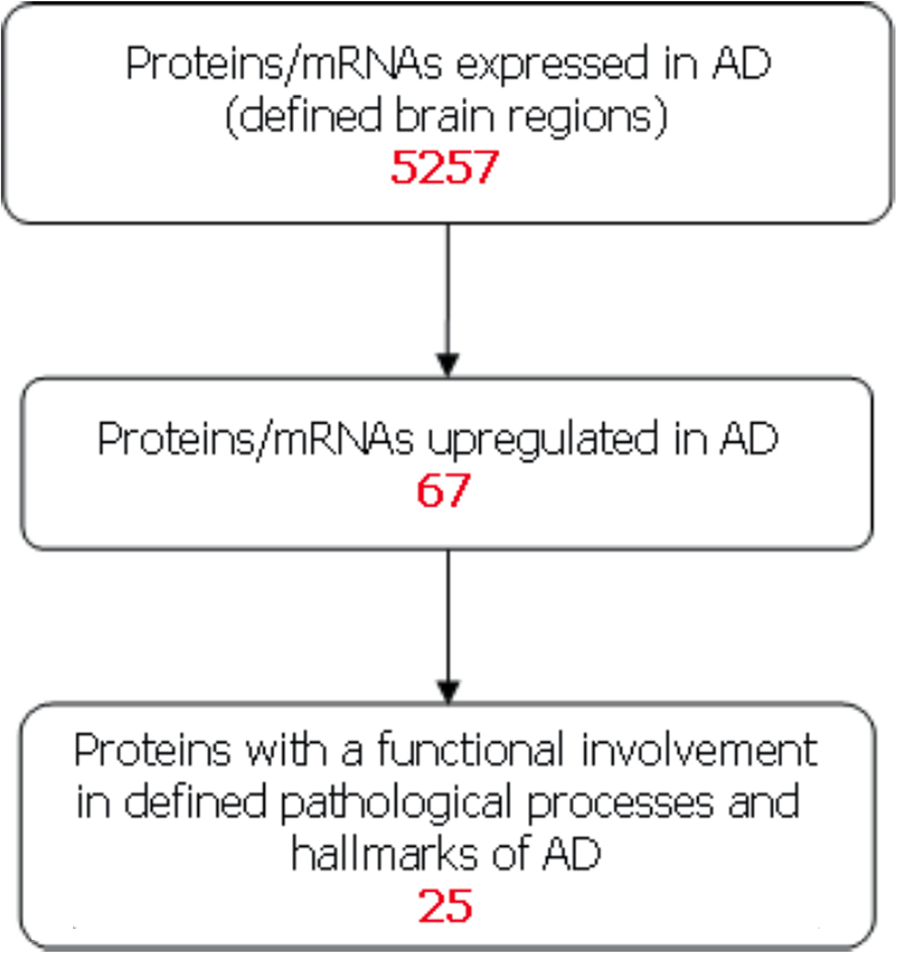
***In silico *workflow to identify candidate AD biomarkers.** The workflow shows the steps used to filter the proteins to a set of potential AD biomarkers. For each step, the number of proteins identified is shown in red.

The use of the resulting IN, which essentially represented a semantically-consistent layer over previously disparate information sources, provided an opportunity to apply simple filtering techniques that in this case led to the derivation of a set of proteins fitting the criteria for a candidate biomarker for AD. According the workflow in Figure 2, the IN was first searched for proteins expressed in brain regions of relevance to AD. This set of proteins was then filtered further, retaining only those with assertional evidence for upregulation in AD. Of those proteins, only those that report assertions highlighting an involvement in the development of the pathological hallmarks of AD were retained. The proteins identified as potential biomarkers by this *in silico* approach were then subjected to an assessment of novelty to see whether relevant publications that discussed the possible use of the biomarkers for AD existed.

### *In vitro* assessment of candidate biomarkers

#### Samples

In order to validate candidate proteins identified in the *in silico* study we utilized samples from the multisite European AddNeuroMed cohort [11]. A total of 300 samples with imaging and clinical data available including 100 each of AD cases, normal elderly controls, and MCI cases and collected from 6 locations across Europe (Kuopio, Finland; London, United Kingdom; Perugia, Italy; Toulouse, France; Lodz, Poland; and Thessaloniki, Greece) were used with all biochemistry assessments being blind to disease status. The AddNeuroMed study including diagnostic process, data and sample collection and curation protocols and imaging processes are described elsewhere [11-13].

#### Imaging

The imaging protocols and analysis of MRI within AddNeuroMed is described elsewhere as noted above. In brief, MR images are subjected to automated analysis following parcellation of the brain in a pipeline that results in over 70 regional cortical volumes and thickness of grey matter variables. Correlational and other analysis of these variables have shown similar diagnostic and predictive qualities as in other, comparable studies such as ADNI [14,15]. Variables that contribute most to diagnostic accuracy include, unsurprisingly those reflecting atrophy in areas of brain known to be most affected by AD including entorhinal cortex and hippocampus.

#### Protein quantization in plasma

We performed semi-quantitative immmunoblotting of diluted (1:50) plasma for two candidate proteins as a validity test process for the *in silico* study. The immunoblotting protocol has been previously described [7] but in brief involved separation by SDS-PAGE, transfer to nitrocellulose, incubation with primary antibody and identification with secondary antibody. Antibodies used in this study included those for ChAt, mouse monoclonal anti-human ChAt antibody (Chemicon Clone 1.B3.9B3) and for PLAUR mouse monoclonal anti-human PLAUR antibody (R&D, CD87, cat.no. MAB807).

Blots were then scanned with Odyssey Infrared Imaging (Li-Cor Bioscience) scanner and analyzed using Odyssey Infrared Imaging System Version 1.2. The Integrated Intensity for each band of the relevant molecular weight was determined and background subtracted.

#### Statistical analysis

The Integrated Intensity value for both ChAt and PLAUR, corrected for protein loading was analyzed using SPSS (v19). Following tests to assess the normality of data distribution (Kolmogorov-Smirnov and Shapiro-Wilk), we first used ANOVA to determine the relationship between protein level and case status and then performed bivariate correlation (Spearman rank correlation) between protein values and brain atrophy measured using MRI.

## Results

### *In silico* discovery of candidate biomarkers

In order to identify potential peripheral AD biomarkers we first performed a consensus building exercise which suggested that an AD biomarker might have the characteristics of being a protein expressed in areas of the brain known to be affected by AD and to be associated with a pathological process relevant to AD and to be differentially expressed in AD. We then utilized text mining and linguistics analysis to construct the ‘AD Biomarker Intelligence Network’ from meta-assertional data derived from all major publically available biological datasets. The workflow described in Figure 2 revealed the following:

• 5,257 proteins and/or mRNAs expressed in AD tissue

• of these, 67 reported evidence for upregulation in AD

• of these, 25 have known associations with a pathological process in AD

These candidate biomarker proteins are listed in Table 3.

**Table 3:** Final set of derived proteins representing candidate biomarker of AD

	**STANDARD PROTEIN SYMBOL**	**COMMON ALIASES**
Alpha 1-antichymotrypsin	SERPINA3	serpin peptidase inhibitor clade A, AACT, ACT
Amyloid beta (A4) precursor protein (protease nexin-II, Alzheimer disease amyloid protein)	APP	AD1 (Alzheimer disease), ABETA (amyloid beta A4 protein)
Apolipoprotein D	APOD	
Apolipoprotein E	apoE	AD2 (Alzheimer disease 2)
B-cell leukemia/lymphoma 2	BCL2	
Beta-site APP-Cleaving Enzyme 1	BACE1	
Butyrylcholinesterase	BCHE	
C-reactive protein, pentraxin-related	CRP	
Choline Acetyltransferase	CHAT	CHOACTase
Clusterin	CLU	APOJ (Apolipoprotein J)
Complement component 1, q subcomponent, beta polypeptide	C1QB	
Estrogen Receptor 1 (alpha)	ESR1	
Glial fibrillary acidic protein	GFAP	
Heat shock 70kD protein 5 (glucose-regulated protein)	HSPA5	
Interleukin 1 beta	IL1B	
Interleukin 6	IL6	IFNB2 (Interferon beta-2)
Matrix Metallopeptidase 9	MMP9	CLG4B (92 kDa gelatinase, 92 kDa type IV collagenase)
Nerve Growth Factor	NGF	NGFB
Nitric Oxide Synthase 2A	NOS2	NOS2A, INOS (Inducible NOS), HEP-NOS (Hepatocyte NOS)
PRKC, apoptosis, WT1, regulator	PAWR	PAR4 (Prostate apoptosis response 4 protein)
Prostaglandin-Endoperoxide Synthase 2	PTGS2	PGHS2, COX2 (Cyclooxygenase 2b)
Transforming Growth Factor, Beta 1	TGFB1	
Transthyretin	TTR	
Tumor Necrosis Factor (TNF superfamily, member 2)	TNF	TNFA, TNF-alfa
Urokinase Plasminogen Activator Receptor	PLAUR	uPAR

### *In vitro* assessment of candidate biomarkers

In order to validate the *in silico* approach to potential biomarker discovery, we chose two proteins not previously suggested as peripheral biomarkers for AD – PLAUR and ChAt – and determined the levels of these proteins in plasma from 240 subjects from the AddNeuroMed cohort, a European AD biomarkers study, using semi-quantitative immunoblotting. Only two proteins not previously associated with AD were tested and these two were chosen partly as one (PLAUR) is a known plasma protein altered in other disease states and one is present in plasma but had not previously been investigated as a biomarker for any condition to our knowledge and partly for serendipitous reasons - antibodies suitable for assay generation were readily available. We aimed first to compare protein levels in research participants with AD, with MCI and normal elderly controls. In addition, as AD has a long prodromal phase and as a consequence many apparently normal elderly people have substantial but occult AD pathology, we also correlated protein levels with cerebral atrophy measured using automated analysis of structural MRI (191 subjects with imaging data) as a quantitative marker of pathological load independent of clinical disease status.

Table 4 shows the diagnostic category, age and gender characteristics of the subjects analyzed together with the results of PLAUR and ChAt analysis. We found PLAUR to be highly significantly different across categories with lowest levels in AD, highest in controls and an intermediate level in MCI (ANOVA, p < 0.001). There was no difference in ChAT comparing the three diagnostic categories. To estimate degree of pathology regardless of diagnostic category we used whole brain volume as a measure of atrophy, derived using automated analysis of structural MRI as previously reported [12,14,15]. For PLAUR we found a very highly significant inverse correlation between whole brain volume and protein levels in plasma. Interestingly the AD group showed a similar direction of effect although this was not significant. For ChAt we also found a highly significant, but in this case positive, correlation between whole brain volume and plasma protein levels in control subjects with again the AD group showing a trend towards the same direction of correlation. In neither case was there any correlation in the MCI group.

**Table 4:** Subject characteristics and PLAUR and ChAt analysis in validation study

						**PLAUR correlation with whole brain volume**			**ChAt correlation with whole brain volume**	
	**N**	**Gender (% F)**	**Age mean (SD)**	MMSE* mean (SD)	PLAUR mean (SD) **	**R**	**p**	ChAt mean (SD) **	**R**	**p**
Controls	82	58	72.8 (7.0)	29 (1.1)	1.63 (0.9)	−0.35	<0.005	0.78 (0.3)	0.34	<0.01
MCI	80	57	74.7 (6.2)	27 (2.2)	0.98 (0.7)	0.14	NS	0.75 (0.3)	0.01	NS
AD	78	69	76.2 (6.4)	21 (4.5)	0.85 (0.3)	−0.2	NS	0.71 (0.3)	0.23	0.06

*MMSE; Mini mental state examination - a cognitive scale (maximum score = 30) used to screen for and assess degree of severity of dementia. A score of less than 24 indicates possible dementia. A score of 10–20 suggests moderate dementia and a score above 20, mild dementia. ** arbitrary units.

## Discussion

The discovery of biomarkers for AD is an increasingly important task – both for early diagnosis and for use in experimental medicine. However, it is a task complicated by at least three major intrinsic difficulties. First the complexity of AD pathology means that identification of candidate markers, beyond the low hanging fruit of AÎ² and tau, is problematical. Second, collection of the optimal peripheral fluid for biomarker identification, CSF, is relatively invasive and unsuitable for repeated measures in elderly people. Third, AD has a prolonged prodrome when apparently normal elderly people harbor considerable pathological load meaning that the conventional case–control design is confounded by pathology in clinically unaffected subjects. Previously we and others have attempted to mitigate the third of these complications by using a design of biomarker discovery where the independent or outcome variable is not clinical diagnosis but an endophenotype of disease such as structural MRI evidence of atrophy [7] or PET evidence of Aβ load [6]. The second of the limitations in biomarkers for AD – the availability of CSF – has prompted many groups to seek markers in other fluids such as plasma. The first of the limitations, the identification of candidates, has been previously attempted by two broad categories of studies; either using candidates based on the researcher’s own understanding of disease or using a data-driven, most often proteomic, approach. Here we combine the use of endophenotypes to complement diagnostic category as an outcome measure, with the use of plasma as a biomarker tissue, with an entirely novel approach to the identification of candidates. This innovation makes use of linguistic and textual analysis to interrogate the entire biomedical knowledge base in the form of all the major publicly available databases to identify candidate markers using a consensus driven set of primary assertions. We accessed the various data sources in 2006, and clearly in a fast moving field data will have changed considerably in the intervening years. Indeed some of the proteins identified in 2006 (such as Transthyretin and Clusterin) had not at that time been considered as biomarkers. However, when the *in vitro* analysis was carried out, data had been provided from our own proteomics studies that these proteins were in fact putative biomarkers. Thus this time lag has inadvertently provided further substantiation of the proof of concept of the *in silico* approach that we discuss here.

The textual analysis of publicly available data sources suggested a total of 25 potential candidate biomarkers. Some of these have previously been identified as potential biomarkers in plasma. For example, using MRI measures of atrophy as an outcome endophenotype we identified and confirmed plasma Clusterin [7] and Transthyretin [16] as measure of severity of disease and using PET measures of amyloid identified apoE protein as the primary correlate in plasma [6]. All these studies used gel based proteomics as the discovery tool and the fact that textual analysis identifies the same proteins before these proteomic studies were performed is a strong indicator of the power of the method. Other promising candidates suggested by textual analysis, and where there is published data suggesting that these proteins are altered either in blood or CSF, include CRP [17-19], Complement factor 1 [20,21], butyrylcholinesterase [22] and BACE1 [23,24]. In all but the case of butyrylcholinesterase, this biomarker data was published after the IN lock-down and hence these biomarker utility data are independent of the IN and act as independent proof of concept.

As the IN identified as potential protein biomarkers proteins previously identified in proteomic studies – without this data entering into this particular network – we were encouraged to attempt further validation in plasma. We chose two proteins - neither previously identified as potential plasma biomarkers to our knowledge - and measured these in over 200 subjects most of whom had as part of the European AddNeuroMed project, automated analysis of structural MRI data available. One of these proteins – PLAUR – was significantly decreased in AD relative to controls with MCI being at an intermediate level. Both PLAUR and ChAT showed a correlation, inverse in the case of PLAUR, with imaging evidence of atrophy in control cases and both showed a smaller and non-significant, but in the same direction, correlation in AD cases. We used semi-quantitative immunoblotting as a screening method as in previous studies as this approach, in contrast to ELISA for example, yields information on degradation products and post translational modifications. In fact the data on these two chosen proteins suggested whole protein correlation with disease state suggesting future biomarker replication and qualification studies, beyond the intention of the present investigation, might progress rapidly to fully quantitative methods.

Urokinase plasminogen activator receptor (PLAUR) is a protein involved in many biological functions including cell signaling [25,26]. By binding urokinase plasminogen activator (uPA), with which it forms an active complex (uPA-PLAUR), it catalyzes the transformation of zymogen plasminogen into the active protein plasmin, a serine protease which degrades fibrin. The receptor is also involved in cell signaling and in chemotaxis, and controls cell adhesion. Increased levels of PLAUR have been previously reported in inflammatory disorders [27] and has been implicated in chemotaxis leading to microglial accumulation in the core of amyloid plaques in brain in transgenic rodent models of AD. AÎ² induces PLAUR [28] and PLAUR is increased in microglia cells of human AD brains and in brains treated with amyloid β peptide [29,30]. The inverse relationship we observe between soluble PLAUR and AD and brain atrophy is noteworthy and might suggest an inverse relationship either between soluble and, functional, membrane bound PLAUR or between central and peripheral PLAUR more generally. An inverse relationship between central amyloid load and peripheral, CSF, amyloid has been previously and extensively noted. The other novel protein association with pathology we identify, ChAt, is a key component of the cholinergic pathway which is severely affected in AD and is the target for the first symptomatic therapies for AD. We observe a relationship between cerebral atrophy and ChAT protein and this may reflect the loss of cholinergic neurons known to occur early in disease process.

In summary we show here that the extraction of data from huge volumes of biological datasets including text based information is possible and that the creation of hypothesis or assertion-driven analysis yields potential biomarkers. As some of these markers have been independently generated using proteomics, and, as here we show at least partial validation of the two markers tested, this finding offers strong support to a text mining approach to biomarker discovery using the ever increasing publically available datasets.

## Competing interests

KCL has protected intellectual property related to biomarkers for AD but unrelated to the work described in this paper, BioWisdom has intellectual property interests in information technology related to the work described in this paper.

## Authors' contributions

ND, JR performed the data-mining and *in silico* analysis. IG, JR-C performed the biomarker assay studies. IG, SL drafted the manuscript. IG, ND, JR, JR-C, HS, AS, JB, SL edited the manuscript. ND, HS, AS, JB, SL participated in study design. HS, IK, MT, BV, CS, PM, L-OW, AS, SL coordinated the clinical study collection of samples and clinical data. All authors read and approved the final manuscript
